# Exposure to Celebrity-Endorsed Small Cigar Promotions and Susceptibility to Use among Young Adult Cigarette Smokers

**DOI:** 10.1155/2013/520286

**Published:** 2013-11-24

**Authors:** Kymberle L. Sterling, Roland S. Moore, Nicole Pitts, Melissa Duong, Kentya H. Ford, Michael P. Eriksen

**Affiliations:** ^1^Institute of Public Health, Georgia State University, 140 Decatur Street, Urban Life Building, Room 878, Atlanta, GA 30303, USA; ^2^Prevention Research Center, Pacific Institute for Research and Evaluation, 1995 University Avenue, SteX 450, Berkeley, CA 94704, USA; ^3^Health Outcomes and Pharmacy Practice Division, The University of Texas College of Pharmacy, 2409 University Avenue PHR Building, 3.209 A 1900, Austin, TX 78712-0127, USA

## Abstract

Small cigar smoking among young adult cigarette smokers may be attributed to their exposure to its advertisements and promotions. We examined the association between exposure to a celebrity music artist's endorsement of a specific brand of small cigars and young adult cigarette smokers' susceptibility to smoking that brand. Venue-based sampling procedures were used to select and survey a random sample of 121 young adult cigarette smokers, aged 18–35. Fourteen percent reported exposure to the artist's endorsement of the small cigar and 45.4% reported an intention to smoke the product in the future. The odds of small cigar smoking susceptibility increased threefold for those who reported exposure to the endorsement compared to those not exposed (OR = 3.64, 95% CI 1.06 to 12.54). Past 30-day small cigar use (OR = 3.30, 95% CI 1.24 to 8.74) and past 30-day cigar use (OR = 5.08, 95% CI 1.23, 21.08) were also associated with susceptibility to smoke a small cigar. An association between young adult cigarette smokers' exposure to the music artist's small cigar endorsement and their susceptibility to smoke small cigars was found. This association underscores the importance of monitoring small cigar promotions geared toward young people and their impact on small cigar product smoking.

## 1. Introduction

In the United States (USA), cigarette consumption has steadily declined during the past decade. However, sales of cigars, particularly little cigars and cigarillos, have markedly increased. Of the three types of cigar products available in the USA, trend data from 1993 to 2006 suggest that the sales of large cigars decreased from 37% to 47%, while the sales of cigarillos increased from 25% to 32% and little cigars increased from 37% to 47% [1]. Increased consumption of small cigars (i.e., little cigars and cigarillos) presents a new challenge for tobacco control researchers. Small cigars are similar to cigarettes in their size, shape, and filtering, are often inhaled, and are flavored. All cigars contain approximately the same toxic and carcinogenic compounds found in cigarettes [2]. Small cigars deliver considerable amounts of nicotine to sustain dependence and increase the carbon monoxide levels of smokers [3, 4]. Small cigar smoking is a public health threat and is not a safer alternative to cigarette smoking.

Small cigar smoking has become increasingly popular among young adults, aged 18–35. Over 65% of young adults have heard about small cigars [5], and 26% report ever use of small cigars [6]. Young adults with a history of tobacco use have an elevated risk for cigar smoking, with recent studies indicating that 12.0–16.0% of young adult cigarette smokers have smoked some cigar variant (cigars, little cigar, or cigarillos) in the past 30 days [6–8]. The increased popularity of small cigars among young adult smokers may be due, in part, to their exposure to advertisements and promotions of small cigar products. Although magazines and point-of-sale advertisements (which accounted for 24.1% and 13.0% of cigar advertising and promotional expenditures in 1999) [9] are traditional avenues for cigar advertisements, tougher restrictions on tobacco advertising may entice the tobacco industry to use creative marketing strategies to promote small cigars. 

In 2012, a celebrity hip-hop music artist, Calvin Broadus Jr., also known as Snoop Dogg, announced his launching of a new brand line of cigarillo products, called Executive Branch. Several widely circulated music magazines and press releases reported that Snoop Dogg planned to “unveil” the new product during his upcoming performances at the 2012 Coachella Music and Arts Festival in Indio, California. The music festival mainly attracts an international audience of adults aged 35 and younger [10, 11]. A recent study by Richardson and colleagues recently documented Snoop Dogg's promotion of Executive Branch small cigars on social media sites, such as Instagram [12]. The tobacco industry historically has relied upon hip-hop and other forms of music to promote smoking, as music can be used as a form of direct, targeted marketing [13]. Hip-hop music has a wide appeal and a varied fan base across different genders, racial/ethnic backgrounds, and nationalities (i.e., USA versus international countries). Endorsements by its celebrity music artists can introduce their youthful fan base (primarily under 30 years old and including many fans under the age of 18) to new tobacco products and smoking behavior. As such, exposure to a hip-hop music artist's endorsement and promotion of a brand-specific small cigar product may be associated with young adults' small cigar smoking behaviors. In 2012, the Surgeon General's report concluded that there is a causal association between exposure to smoking in the movies and youth smoking. The report also concludes that traditional advertising causes youth smoking [14, 15]. To our knowledge no study has examined the association between small cigar smoking behavior and exposure to a celebrity music artist's advertisement and promotion of small cigars. We surveyed a random sample of young adult cigarette smokers who attended the Coachella festival in Indio, California, to assess their exposure to Snoop Dogg's endorsement and advertisement for Executive Branch small cigars. We focused on young adult cigarette smokers because extant evidence suggests that they may be considered at high risk for small cigar use [6, 7, 16]. We hypothesized that young adults who reported exposure to the artist's small cigar advertisements, compared to those who did not report exposure, would have greater susceptibility or intention to smoke Executive Branch small cigars in the future. Our hypothesis is supported by ample evidence that has reported that exposure to cigarette smoking advertisements is associated with a susceptibility to smoke cigarettes [17–20]. Our rationale for use of intention to smoke small cigars is that it is a proximal predictor of smoking behavior [21] and is the most appropriate outcome to assess in a cross-sectional design.

## 2. Methods

### 2.1. Participants and Sampling Frame Construction

Eligible participants were young adults, aged 18–35, who attended the Coachella festival and reported smoking at least one cigarette in the past 30 days. Approximately 85,000 concertgoers attended the festival per day over a three-day period [22]. According to a small survey of concertgoers (*n* = 238) posted on the festival's webpage (http://www.coachella.com), 31.5% were aged 25–29 years, 29.8% were aged 20–24 years, and 12.2% were aged 30–34 years old. Venue-based sampling procedures [23], in which the venue is the primary sampling unit, were used to select and survey eligible young adults at the festival. 

To construct our sampling frame, the study team (K.S. and N.P.) utilized ethnographic sampling techniques to survey the area and identify locations within the festival where young adult smokers would gather or pass through. We identified three locations within the venue that fit this criterion: two “beer gardens” where festival attendees purchased and consumed alcoholic beverages and a smoking lounge. Each location was restricted to individuals aged 18 and older. Within the smoking lounge, we implemented area-based sampling and approached each individual for inclusion into the survey. For the two “beer gardens,” we identified young adults who were smoking cigarettes and randomly selected them by systematically intercepting every third cigarette smoking attendee that crossed a predetermined point. 

### 2.2. Procedures

The two study team members collected data from attendees over a two-day period from festival open to close. Upon interception, each team member approached festival attendees, gave them a short description about the study, and obtained verbal consent. If the attendee refused, the study team member noted their refusal and continued the process with the next random participant. Festival attendees who appeared to be visibly impaired by alcohol or other substances were not approached. The study team determined the eligibility status of festival attendees that were intercepted and who were willing to participate using a brief screener that verified the attendant's age and cigarette smoking status. Attendees who did not meet the eligibility criteria were not surveyed.

Over the two-day data collection period, a total of 275 participants were approached. Of those 126 (45.8%) refusals were noted and 6 (2.2%) participants did not meet study eligibility criteria. Data were lost for 22 respondents due to a synchronization error between the server and the device and could not be retrieved. Thus survey responses were collected and retained for 121 young adults.

### 2.3. Study Sample Description

 The majority of the 121 respondents were male (55.4%) and between the ages of 18 and 30 years old (88.0%). Regarding race/ethnicity, most respondents were white (75.2%) and lived in a country outside of the USA (52.4%). Overall, 70% reported past 30-day nonmentholated cigarette smoking, while 36.4% smoked menthol cigarettes. 

### 2.4. Measures

Survey items were entered in a survey software app, iSurveySoft (http://www.isurveysoft.com), and were uploaded to the handheld device (iPod touch 5th generation). The brief survey consisted of 21 items including demographic information, smoking history and current tobacco use, and exposure to small cigar advertisements. 

#### 2.4.1. Demographic Information

 Respondents were asked about their age, gender, race/ethnicity, and country and state of residence. Age categories included <18 years old, 18–24 years old, 25–30 years old, and 31 years or older. Racial/ethnic categories included Asian/Pacific Islander, American Indian/Alaskan Native, Black/African-American, Hispanic, White, and other. Respondents were allowed to select one or more of the racial/ethnic categories. 

#### 2.4.2. Smoking-Related Variables

 Respondents were asked if they had used any of the following tobacco products in the past 30 days: cigarettes, nonmentholated (i.e., Marlboro), mentholated cigarettes (i.e., Newports), cigars, and small cigars (e.g., Black & Mild, Swisher Sweets). The response categories for each included “yes” or “no”. Brand names were provided for each tobacco product to help respondents better recognize the tobacco product [24]. Respondents were allowed to select one or more of the tobacco products if they used them in the past 30 days. Those who selected “yes” for the past 30-day use of any of the tobacco products were classified as current users. 

#### 2.4.3. Susceptibility to Smoke Small Cigars in the Future

 Respondents were asked two questions: (1) if they intended to smoke Executive Branch small cigars and (2) if they intended to smoke any other small cigar in the future. Response categories for both questions were “yes” or “no.”

#### 2.4.4. Exposure to Small Cigar Advertisements

We assessed festival attendees' exposure to (1) the Snoop Dogg's advertisements of the Executive Branch small cigars and (2) other advertisements for other small cigar products (e.g., Swisher Sweets). Brand names were provided to assist the respondents with recall. Response categories for both questions were “yes” or “no.” If a respondent reported exposure to the product, they were asked to recall where they had seen the advertisement. Response categories included in a newspaper, in a magazine, on the Internet, on social media sites (e.g., Facebook, Twitter, etc.), and other. 

#### 2.4.5. Urge to Smoke Small Cigars

Urge to smoke Executive Branch small cigars was assessed by asking respondents how much they wanted to smoke the product after exposure to Snoop Dogg's advertisement. The urge to smoke variable is similar to the one used by Sargent and colleagues [25]. A four-point response category was used to assess urge to smoke and included 1 = strongly agree to 4 = strongly disagree. We assessed exposure to and urge to smoke other small cigar products with similar questionnaire items, by replacing the phrasing “celebrity-endorsed small cigar product” with “other small cigar products.” 

### 2.5. Analysis

Respondent demographics, smoking-related behaviors, advertising exposure, and urge to smoke variables were explored using descriptive statistics. We assessed the prevalence of the past 30-day small cigar smoking among our sample of young adult cigarette smokers. To understand the demographic and tobacco use characteristics of small cigar users in our sample, we conducted descriptive analyses comparing survey responses from those young adult cigarette smokers who reported past 30-day use of small cigars to those who did not report use. Descriptive statistics were also used to assess exposure to small cigar advertisements (celebrity-endorsed and other) and the urge to smoke small cigars after viewing the ads. Bivariate and multivariate logistic regression analyses were conducted to assess the associations between small cigar smokers and nonusers and the demographic, smoking-related, exposure, and urge to smoke variables. The dependent variable for the bivariate and multivariate analysis was small cigar smoking.

## 3. Results

### 3.1. Small Cigar Smoking Behavior and Demographic Characteristics

Of the 121 young adult cigarette smokers in our sample, 25.6% reported smoking small cigars at least once in the past 30 days.  Table 1 shows the distribution of demographic characteristics by small cigar smoking status. The majority of small cigar smokers in our sample were male and aged 18–24 years old, though a sizeable proportion were aged 25–30 years old. Although most small cigar smokers in our sample were White, significantly more African-Americans reported smoking a small cigar in the past 30 days than not smoking a small cigar (*P* = 0.001). Notably, over half of the small cigar smokers in our sample lived in a country outside of the United States.

### 3.2. Concurrent Tobacco Use among Small Cigar Smokers

 Small cigar smokers (*N* = 31) in our sample reported concomitant use of other tobacco products. Over two-thirds (71.0%) of small cigar smokers reported smoking nonmentholated cigarettes in the past 30 days, while one-third (32.3%) reported using mentholated cigarettes. Small cigar smokers also reported current cigar smoking, with 19.4% reporting smoking cigars at least once in the past 30 days.

### 3.3. Exposure to Small Cigar Advertisements and Urge to Smoke

Approximately 14.0% of respondents reported seeing Snoop Dogg's advertisement of Executive Branch small cigars. The majority of respondents (33.3%) reportedly saw these advertisements on the Internet, while 27.8% reported seeing them on social media sites, such as Facebook or Instagram. Of the respondents who were exposed to the Executive Branch advertisements, 82.4% said seeing the advertisement made them want to try the product.

Approximately 60.2% of our respondents had been exposed to other small cigars advertisements. Respondents reported seeing advertisements for the other small cigars in magazines (35.1%), on the Internet (23.0%), in convenience stores or bodegas (12.4%), in smoke shops (6.6%), and on social media sites (5.4%). Over half of respondents (55.1%) wanted to try the small cigars after being exposed to the advertisements. 

### 3.4. Susceptibility to Smoke Executive Branch Small Cigars

 Forty-five percent (45.4%) of 121 young adult cigarette smokers in our sample reported an intention to smoke Executive Branch small cigars in the future; of these, 40.9% were current small cigar smokers. Bivariate and multivariate logistic regression analyses were conducted to assess whether the demographic and smoking-related variables and the exposure to Snoop Dogg's Executive Branch small cigar advertisements were associated with susceptibility to smoke the product in the future. Though demographic variables were not associated with susceptibility, bivariate analyses found that past 30-day cigar (OR = 4.90, 95% CI 1.26, 19.13, *P* = 0.02) and small cigar (OR = 2.98, 95% CI 1.19, 7.42, *P* = 0.02) smoking were significantly associated with an intention to smoke Executive Branch small cigars. Exposure to Snoop Dogg's advertisements was marginally associated with intention to smoke Executive Branch small cigars (*P* = 0.08). Given the exploratory nature of our study, variables from the bivariate analysis that were significant at *P* < 0.10 were included in the multivariate analysis.  Table 2 shows the results of our multivariate model. Respondents who reported current cigar smoking were five times more likely and those who smoked small cigars were three times more likely to be susceptible to smoking Executive Branch small cigars. Respondents who reported seeing the artist's advertisement for Executive Branch small cigars were three times more likely to be susceptible to smoking those small cigars than those who were not exposed to the advertisement. 

### 3.5. Susceptibility to Smoke Other Small Cigars in the Future

Over half (53.7%) of our respondents intended to smoke other small cigars in the future; 41.5% were already current small cigar smokers. Bivariate analyses found that past 30-day cigarette (*P* = 0.06), cigar (OR = 3.75, 95% CI 1.00, 14.00, *P* < 0.05), and small cigar smoking (OR = 16.34, 95% CI 3.65, 73.18, *P* < 0.001) were significantly associated and exposure to other small cigar advertisements (*P* = 0.06) was marginally associated with the susceptibility to smoke other small cigars.  Table 2 shows the results of our multivariate model for susceptibility to other small cigars. Past 30-day cigar and small cigar smoking were significantly associated with susceptibility to smoke small cigars in the future. Exposure to other small cigar advertisements was not significantly associated with susceptibility to smoke other small cigar products, however.

## 4. Discussion

Small cigar smoking is a growing public health concern among young adults, particularly for those with a history of tobacco use. Among our sample of young adult cigarette smokers, over 25% concurrently smoked small cigars in the past 30 days. Our finding is consistent with that of prior studies [6, 7]. Concurrent use of both products is a public health concern, as it may increase young adults' risk for developing nicotine dependence [2, 26], may lead to an escalation of tobacco use, and may make it difficult to quit smoking and achieve long-term abstinence.

Our preliminary findings suggest that exposure to small cigar advertisements may explain, in part, young adults' awareness of small cigars. Over 60% of our respondents reported exposure to other small cigar advertisements, and 14% reported exposure to Snoop Dogg's Executive Branch small cigar advertisement. Although traditional outlets such as magazines and convenience stores were also sources of advertisement exposure, respondents reported exposure to small cigar advertisements on the Internet, particularly on social media sites. This is consistent with growing body of evidence that has found protobacco images and references [27–29], in particular small cigar smoking [12, 30] on Internet social media sites. 

 We have little evidence to support the claim that the tobacco industry paid for or supported the hip-hop artist's endorsement of this small cigar product. However, the Federal Trade Commission Report of 1999 documented the cigar industry's payments to celebrities for their endorsements, appearances, and cigar product placements. It is well documented that cigarette smoking has been promoted through rap and hip-hop music [15, 31] and through music-themed campaigns (i.e., the Kool Mixx campaign by Brown & Williamson [13], and concerts and festivals such as Camel's “Smooth Moves” and “Speakeasy” tours) [32]. Rap and hip-hop music genres have a wide reach. Hip-hop artists appeal to individuals across gender, nationalities (USA versus international), racial/ethnic backgrounds and ages (although as noted previously, to youth in particular). These artists are often viewed as trendsetters. Taken together, the artists' endorsement of a cigar product may establish brand loyalty and influence the smoking behaviors of large segments of their fans. Perhaps the use of celebrity rap and hip-hop artists to promote small cigar products and smoking is a strategy that is being used by the cigar industry to circumvent tobacco advertising restrictions. 

Exposure to the hip-hop artist's small cigar advertisements appeared to influence small cigar smoking susceptibility among the young adult cigarette smokers in our sample. Those who reported exposure to the artist's Executive Branch small cigar advertisements were three times more likely to intend to smoke these small cigars in the future. Notably, over 40% of those who reported an intention to use the Executive Branch small cigars were already past 30-day small cigar smokers. Although not previously documented for music entertainment, our findings are consistent with studies that found an association between exposure to prosmoking depictions in films and smoking behavior among adolescents [15]. Future studies that examine the association between exposure to small cigar advertisements and intention to smoke among other young adult smoking samples are needed to substantiate our findings. 

An important limitation of this study is its generalizability, as the survey sample largely consisted of a sample of young adult festival attendees in the United States. Findings from this study may not generalize to other young adult populations. While we broadly assessed other small cigars, this study specifically examined exposure to Snoop Dogg's advertisements and promotions of Executive Branch small cigars. As such, our findings may not generalize to other small cigar brands. We used brand-specific items to estimate small cigar use among our respondents. Though these items may estimate small cigar use more accurately [24], their use may also limit comparability to studies that used other measures to capture small cigar use (i.e., single “catch all” question assessing cigar product use). Finally, the items that assessed small cigar use in our study did not specifically ask if participants were smoking tobacco in the small cigar. Despite the use of this item to measure small cigar smoking in other studies [8], its use may bias findings because it may also capture use of marijuana. Thus, our findings should be interpreted with caution.

### 4.1. Conclusion

Our study documented young adult smokers' exposure to a celebrity hip-hop artist's advertising of Executive Branch small cigars. We also provided preliminary evidence of an association between exposure to a celebrity artist's small cigar promotion and susceptibility to smoke the brand-specific small cigar. Our findings indicate that celebrity endorsement is a potentially important source of marketing small cigar products to young adults and should be monitored. 

## Figures and Tables

**Table 1:** Demographic information of young adult cigarette smokers attending the festival.

	Past 30-day small cigar smoker (*N* = 31, %)	Nonsmall cigar smokers (*N* = 90, %)	Total sample (*N* = 121, %)	*P* value
Country of residence				
United States	58.1	55.6	47.6	
Outside of United States	41.9	44.4	52.4	
Age				
18–24 years	51.6	48.9	42.6	
25–30 years	48.4	51.1	43.4	
≥31 years	0.0	0.0	14.0	
Sex (% male)	58.1	54.4	55.4	
Race (respondents were able to select more than one, thus total >100%)				
Asian/Pacific Islander	9.7	6.7	7.4	
American Indian/Alaskan Native	3.2	1.1	1.2	
Black	22.6	5.6	9.9	**
Hispanic	13.3	12.9	13.2	
White	67.7	77.8	75.2	
Other	3.2	1.1	3.4	

*P < .05, **P < .01, ***P <.001.

**Table 2:** Multivariate logistic regression analyses predicting small cigar susceptibility.

Predictor	OR (95% CI) for intention to smoke celebrity endorsed small cigar	*P *	OR (95% CI) for intention to smoke other small cigars	*P *
Current cigarette use	—		2.55 (0.94, 6.89)	
Current cigar use	5.08 (1.23, 21.08)	*	4.22 (1.05, 16.93)	*
Current small cigar use	3.30 (1.24, 8.74)	*	17.55 (3.87, 79.69)	***
Exposure to celebrity-endorsed small cigar advertisements	3.64 (1.06, 12.54)	*	1.99 (0.81, 4.95)	

*P < .05, **P < .01, ***P <.001.
